# Development of Biodegradable Whey-Based Laminate Functionalised by Chitosan–Natural Extract Formulations

**DOI:** 10.3390/ijms21103668

**Published:** 2020-05-22

**Authors:** Sanja Potrč, Lidija Fras Zemljič, Meta Sterniša, Sonja Smole Možina, Olivija Plohl

**Affiliations:** 1 Laboratory for Characterization and Processing of Polymers, Faculty of Mechanical Engineering, University of Maribor, Smetanova 17, SI-2000 Maribor, Slovenia; olivija.plohl@um.si; 2 Faculty of Chemistry and Chemical Engineering, University of Maribor, Smetanova 17, SI-2000 Maribor, Slovenia; 3 Department of Food Science and Technology, Biotechnical Faculty, University of Ljubljana, Jamnikarjeva 101, SI-1000 Ljubljana, Slovenia; meta.sternisa@bf.uni-lj.si (M.S.); sonja.smole-mozina@bf.uni-lj.si (S.S.M.)

**Keywords:** whey laminate, functionalization, active packaging, chitosan, plant extracts, antimicrobial, antioxidant

## Abstract

In this research, antimicrobial polysaccharide chitosan and natural extracts were used as surface coating of a plastic laminate with an integrated whey layer on the inside. The aim was to establish the biodegradable and active concept of packaging laminates. For this purpose, chitosan nanoparticles (CSNPs) with embedded rosemary or cinnamon extracts were synthesised and characterised. Additionally, a whey-based laminate was functionalised: i) chitosan macromolecular solution was applied as first layer and ii) cinnamon or rosemary extracts encapsulated in CSNPs were applied as upper layer (layer wise deposition). Such functionalised whey-based laminate was physicochemically characterized in terms of elemental surface composition, wettability, morphology and oxygen permeability. The antimicrobial activity was tested against *Staphylococcus aureus, Escherichia coli, Aspergillus flavus* and *Penicillium verrucosum*. The antioxidant properties were determined using the ABTS assay. It could be shown that after functionalization of the films with the above-mentioned strategy, the wettability was improved. Furthermore, such whey-based laminates still show excellent barrier properties, good antimicrobial activity and a remarkable antioxidative activity. In addition to the improved biodegradability, this type of lamination could also have a positive effect on the shelf-life of products packaged in such structured films.

## 1. Introduction

It is estimated that around 12 million tonnes of plastic waste per year are released from waste management systems and released into the environment worldwide, with packaging materials in the majority, consumer demand and government legislation worldwide being the driving forces behind the sustainable packaging agenda [1]. The environmental awareness of a growing consumer population is fuelling demand for sustainability and the reduction of the environmental impact of packaging [2,3,4]. The reuse and recycling of plastics, especially for some applications such as packaging, remains very low, so it is extremely important to establish a biodegradable concept alongside functional packaging [5,6,7]. While most progress has been made in reducing the environmental footprint (both in terms of biodegradability and the origin of packaging raw materials) and packaging costs, it is recognized in the food packaging industry that until recently there have been few commercial examples of the benefits of packaging functionality for the consumer [8]. Specifically, there is a recognized need for so-called eco-innovative functional food packaging materials [9], which can directly benefit consumers through improved shelf-life, monitoring of food quality, and assurance of food safety, while at the same time maintaining a reduced environmental footprint compared to standard food packaging. Thus, besides biodegradability as a first priority, these packaging materials should have active and/or intelligent functionality. Active systems can be successfully used to extend the shelf-life of processed foods and can be categorized into adsorption and release systems [10]. On the other hand, intelligent packaging is able to monitor the condition of packaged food or the environment by providing information about different factors during transportation and storage, including time-temperature indicators, gas detectors, and freshness and/or ripening indicators [11,12]. Advances in nanotechnology and nanomaterials have enabled the efficient development of new active and intelligent packaging systems, which can improve the freshness, and shelf-life of food, while monitoring the entire retail system (storage conditions from the place of production to the place of consumption by the final consumer) [13]. With these developments, eco-innovative food packaging materials are rapidly becoming a strategic target of the European food sector and thus also of a research community [14]. The food industry is one of the applications, where coextruded laminate plastic films are becoming increasingly important and widely used [15]. They are mainly used for packaging of products such as meats, cut vegetables and fresh pasta to extend the shelf-life of the goods [16]. Nowadays, commercial multilayer packaging materials consist of a number of layers of different polymers [17,18]. In most applications, they are assembled in such way that the outer layers consist of cheap polymers with good mechanical and water barrier properties (polyethylene (PE), polypropylene (PP), polystyrene (PS)). On the contrary, the inside of the packaging materials consists of more expensive materials, which are of a great importance in terms of gas-barrier properties (polyvinylidene chloride (PVdC), ethylene vinyl alcohol (EVOH), polyethylene terephthalate (PET)). However, the problem with such multilayered laminates is that they are not biodegradable. Moreover, the conventional petroleum-based plastics not only consume non-renewable feedstocks, but also have negative environmental impacts, mainly due to waste disposal [19]. Thus, there is a big need for the development of new biodegradable laminate films for food packaging applications. These new materials mainly include laminates based on modified starch and polylactic acid (PLA) [20,21], which are anticipated to present good water and gas-barrier properties; they are also easily extruded and processed, and are eventually (bio)degraded at the end of the product life [16]. Whey films as part of laminates have also been developed with a good prospect [22]. Although recent research has focused on the composition of laminates with emphasis on the effects on biodegradability, there is still very little research on the development of functional laminates.

Reviewed publications mostly pointed out the development of biodegradable and functional packaging materials on their own, rather than as an integrated part of laminates. Among those concepts, essential oils represent an interesting ingredient for biodegradable food packaging, mainly due to their natural origin and their functional (antioxidant/antimicrobial) properties, which make it possible to obtain active materials designed to prolong shelf-life and add value to the product [23,24]. It was found that the incorporation of essential oils affects the continuity of the polymer matrix, leading to physical changes, depending on the specific interactions between the polymer-oil components [25]. Generally, the film structure is weakened by the oil addition, whereas the water barrier properties are improved, and the transparency is reduced. Essential oils may provide the films with antioxidant but also limited antimicrobial properties (to low spectrum of microorganisms) [26]. In the study by Cabello et al., PLA films that contain different concentrations of essential oil from *Origanum vulgare L. virens* (OEO) were developed [27]. The effectiveness of this new active packaging was checked for use in ready-to-eat salads. A plasticizing effect was observed when OEO was incorporated in PLA films. The rest of the mechanical and physical properties of developed films did change a bit when OEO was included in the film. An antioxidant effect was recorded only for films containing the highest percentages of the active agent (5% and 10%). Moreover, antimicrobial activity was observed only against yeast and moulds, where 5% and 10% of OEO were the most effective. In another study, the antimicrobial properties of whey protein isolate films with different amount of oregano, rosemary and garlic essential oils were tested against *Escherichia coli, Staphylococcus aureus* and others [28]. It has been shown by their study that the most effective against tested bacteria was film containing garlic essential oil, while the use of rosemary essential oil in whey protein isolate film did not exhibit any antimicrobial activity.

It became clear that when essential oils are incorporated into biodegradable packaging mats/films, high concentrations are needed, and finally some disturbances on the final properties as well as limited bioactivity can be observed. In this context, the combination of these essential oils with biopolymers, such as polysaccharide chitosan, is recommended to improve the impact of chitosan and natural extract on the physicochemical and bioactive properties of packaging materials [29,30]. Chitosan as an antimicrobial polysaccharide coating in combination with oregano essential oil was studied also on dry fermented sausage [31].

Moreover, in the study Khanjari et al., pure chitosan and whey protein films alone or incorporated with nanoliposomal garlic essential oil did not show any inhibitory effects on tested microorganisms [32]. Incorporation of 2% or higher concentrations of garlic essential oil in the chitosan solution showed the antibacterial activity of the films against all tested microorganisms. Incorporation of the whey protein film solution with 3% or higher concentrations of garlic essential oil also showed good antibacterial activity of these films against all microorganisms tested. Furthermore, the results revealed that the inhibition of *S. aureus* and *Listeria monocytogenes* was improved by the additive effect of chitosan and garlic essential oils. Shoja et al. have reported on the antimicrobial activity of chitosan and edible whey protein films that were incorporated with different concentrations of essential oil [33]. They found out that these edible films with essential oil have an extremely good inhibitory effect against common food-borne pathogenic bacteria and can be used as an active packaging ingredient in order to prolong the shelf-life of food and to guarantee the food safety. Furthermore, the synergistic effect of chitosan and zein nanoparticles on the barrier properties of the whey protein isolate film was studied [34]. Glycerol plasticized whey protein isolate nanocomposites were produced with incorporation of chitosan and zein particles. The moisture barrier properties of the whey protein isolate nanocomposite films were improved with the addition of particles [34]. In other study, natural bio-based zein films were prepared by incorporating cinnamon essential oil (CEO) and chitosan nanoparticles (CSNPs) at 2% and 4% (*w/w*) amounts, respectively, in order to provide mechanical and antimicrobial functionalities. The antimicrobial properties were investigated against *Escherichia coli* and *Staphylococcus aureus*, observing that their growth was considerably inhibited by the addition of CEO alone and in combination with CSNPs in zein films, while CSNPs-loaded zein film had no significant effect on the growth of microorganisms [35].

Furthermore, despite several advantages of using whey protein layers, it is well known that the protein layers cannot be used alone in the packaging applications due to their brittleness, so they are often used in combination with other polymers in a sandwich or laminate structure [36]. Therefore, whey-based layers in multilayered films based on PE and PET have been proposed, and it has been outlined that the whey layer is not only biodegradable, but can also achieve superior barrier properties compared to other bioplastics. Additionally, it has also been stated that it behaves similarly to synthetic barrier layers, e.g., EVOH [22,36,37]. Therefore, in this study the whey protein-based layer as part of the laminate structure was used for further modifications to introduce the active concept on the inside in contact with food. In this way, the laminate can extend the shelf-life of food and make it safer.

Although the above-mentioned contributions report on the production of whey protein-based films in combination with either essential oils or the separate use of chitosan nanoparticles, to the best of our knowledge, there is no report on the synergistic use of polyphenols incorporated into chitosan nanoparticles and then attached to a whey-based laminate previously coated with a macromolecular solution only (two-layer coatings). The macromolecular solution of chitosan enables better adhesion of chitosan nanoparticles with the embedded extract, and thus improved stability of the polyphenols on the film surface. In addition, our strategy includes coatings of nanoparticles dispersed in the remaining chitosan and extract solution, based on the idea that an increase in stability can be achieved by simply embedding the nanoparticles in another macromolecular layer.

As such, this approach shows a clear and significant advance when compared to the other state-of-the-art investigations. Thus, the aim of the present study was to evaluate the effects of this two-layered and nanostructured surface coating on the physical, chemical, antioxidative and antimicrobial properties of whey-based laminates, as an active film applied on the inner side of plastic laminates for food packaging.

## 2. Results and Discussion

### 2.1. Characterization of CSNPs Colloidal Formulations with Embedded Extracts

**Figure 1:**
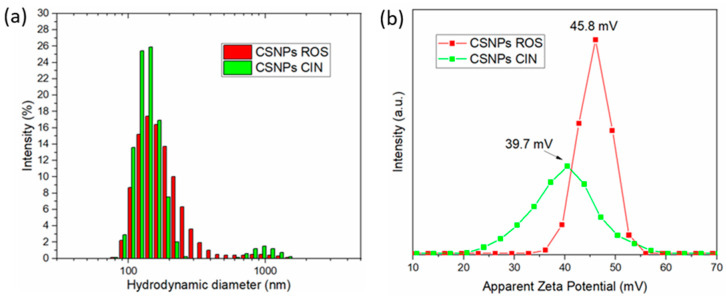
Hydrodynamic diameter and size distribution by number of chitosan nanoparticles (CSNPs) with embedded extracts is shown in (**a**), while their corresponding zeta potential (ZP) distribution at pH 4 is given in (**b**).

After using the ionic gelation procedure for preparing CSNPs with embedded extracts, the electrokinetic properties in terms of hydrodynamic diameter and zeta potential (ZP) of prepared dispersions were verified using dynamic and electrokinetic light scattering of both aqueous dispersions at pH 4. The results obtained for rosemary and cinnamon extracts (ROS and CIN) encapsulated in chitosan nanoparticles are shown in Figure 1. The hydrodynamic diameter distribution of both extract-embedded CSNP dispersions revealed quite a broad size distribution, whereas a larger size distribution with less fraction of agglomerates can be identified for the CSNPs ROS (Figure 1a). For the latter, the majority of the particles were in the size range between 80–500 nm, while some particles with the size exciding the 1 μm could be also seen. This can be attributed to the particle agglomerating to a small extent, which was proved by the polydispersity index (PDI) being higher than 0.5. In fact, the PDIs for CSNPs ROS and CSNPs CIN were 0.9 and 1, respectively. Although the CSNPs CIN (Figure 1a, labelled in green) show similar size distribution characteristics with the ROS-based particles, some differences can be seen that are attributed to a higher intensity of particles with a hydrodynamic diameter of around 150 nm and with the narrower size distribution in the particle range 80–260 nm. Despite this, the intensity of the larger particles in terms of microns is a little bit higher. Nevertheless, both of the prepared dispersions showed suitable characteristics in terms of nanosize, which is reflected in the larger active area and absence of the larger fraction of agglomerates for their further application as antioxidant and antimicrobial effective layers [38].

ZP, as an indicator of the prepared dispersion stability, was quite high in the case of both prepared dispersions, and from a theoretic point of view, it exceeded the absolute value of 30 mV that can be regarded as a stable dispersion with minimum sedimentation [39]. The ZP of ROS and CIN-embedded CSNPs corresponded to 45.8 and 39.7 mV, respectively (Figure 1b). The high positive value of the ZP in both cases is the consequence of protonated amino groups from CSNPs at pH 4 that exhibit highly positive charge, and at the same time indicates successful incorporation of extracts into the interior of the CSNPs and the accessibility of the CS amino groups [40]. Nevertheless, some polyphenols may have been also present at the surface of the CSNPs, and this may be the reason why the CSNPs CIN exhibit a smaller absolute ZP in comparison to the CSNPs ROS. Subsequently, the ZP results are in agreement with the hydrodynamic diameter and size distribution of both dispersions.

**Figure 2:**
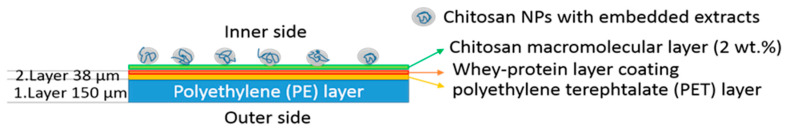
The scheme of multilayer whey protein-based film.

The results of the electrokinetic properties and particle size of CSNP colloidal formulations with embedded extracts showed a successful formation of particles in the nano range with a high positive charge. As such, they were deposited on the inside of the whey-based laminate, as shown schematically in Figure 2, where the structure of the entire protein-based laminate is shown. PE and PET layer were interconnected using an adhesive and the inner side is supposed to be in contact with the food. Following this, the modified whey-based laminate prepared in this way was further subjected to various physicochemical characterizations, as discussed below.

### 2.2. ATR-FTIR Spectroscopy

**Figure 3:**
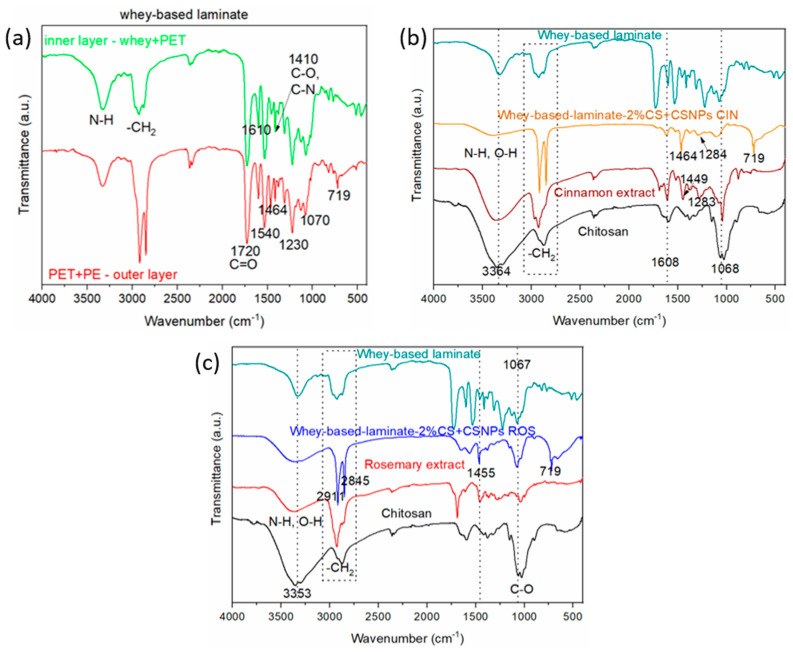
Attenuated Total Reflection–Fourier Transform Infrared (ATR-FTIR) spectra of different layers of whey-based laminate film structure is shown in (**a**); IR spectra of pristine, extracts, chitosan and the modified whey-based laminate film for the 2%CS+CSNPs CIN is shown in (**b**); and the whey-based laminate film with the deposited 2%CS+CSNPs ROS is shown in (**c**).

The presence of the different functional groups of the whey-based laminate structure, as well as different functionalization steps of whey-based laminate functionalised with the 2%CS + CSNPs-embedded extract was followed by the Attenuated Total Reflection–Fourier Transform Infrared (ATR-FTIR) spectroscopy (Figure 3). To clarify and to confirm the whey-based laminate structure (Figure 2), the whey-based laminate was unfolded into two layers as whey + PET (“2.Layer” in Figure 2) and PET + PE (“1.Layer” in Figure 2) and corresponding infrared (IR) spectra of both layers in the direction from whey towards PE (from “2. Layer” to “1. Layer” as shown in Figure 2) are presented in Figure 3a. Due to the strong interconnection among different layers, it was impossible to separate the whey and PET layers, and some traces of the PET layer remained on the last PE layer. In the case of the pristine whey-based laminate film with the innermost layer corresponding to the whey and PET layer, the bands at 2911 cm^−1^ can be assigned to the asymmetrical stretch CH_2_, and the band at 2845 cm^−1^ to the symmetrical stretch CH_2_. As already explained above, these two layers could not be separated completely and due to the depth of IR light in terms of tens of μm, the typical functional groups of both layers can be observed.The same scenario is also observed in the case of the PET+PE layer. 

In the most inner layer of the whey-based laminate (Figure 3a, labelled in green) structure, it is possible to observe that the spectrum of whey is easily distinguishable from that of the PET because of the intense band attributable to N-H stretching and the typical amine I and amide II band at 1610 and 1540 cm^−1^ due to the C=O stretching and N-H bending, combined with C-N stretching (protein structure). The spectrum of PET shows typical C=O stretching peak at 1720 cm^−1^ and the C-O and C-O-C stretching bands at 1230 and 1070 cm^−1^, respectively [41]. The bands located at 1464 and 719 cm^−1^ correspond to bending and rocking deformation of the PE layer in the PET+PE laminate structure. The latter appears to be more broadened when compared to pure PE [40,42] and can be attributed to the effect of the laminate structure and to the contribution of other functional groups originating from the most inner layer of laminate structure (Figure 3a).

The outer layer of laminate structure (Figure 2) was also monitored by ATR-FTIR (Figure 3a, spectra is labelled in red), and the spectra represent the presence of the typical functional groups of PET and PE as expected for this part of the laminate, and confirm the laminate layer composition. This is due to the fact that the spectra were measured on the inner side of the outer layer which is still in contact with the “1. Layer” of the whey + PET layer, therefore the occurrence of some whey layers can also be found here.

The pure chitosan spectrum (labelled in black in the IR graphs in Figure 3b,c) represents the ATR-FTIR spectra of pure chitosan powder, where typical bands can be observed, such as N-H and O-H stretching at the wavenumber of around 3364 cm^−1^. At 1657 cm^−1^, left acylated groups are seen (C=O stretching of the amide I) and N-H bending, together with C-N stretching vibrations at 1588 cm^−1^, whereas the band at 1150 cm^−1^ can be attributed to the C-O-C bonds. The bands located at a wavenumber of around 1066 cm^−1^ are assigned to C-O stretching of glycosidic bonds [42].

The characteristic fingerprints for the cinnamon were mostly present in the range of 1800–600 cm^−1^ and are shown in Figure 3b (labelled in brown) [43]. The peak around 1608 cm^−1^ corresponds to the stretching vibration of an aldehyde carbonyl C=O, and also the peak at 1449 cm^−1^ is typical for the alcohol C-OH. The cinnamon peaks at around 1000 cm^−1^ and 1068 cm^−1^ are attributed to the stretching vibrations of C-O and C-OH deformation vibration. The peak at 1283 cm^−1^ is attributed to the CH_2_ alkanes that face the swing and the aromatic ring C-H for the in-plane bending absorption [43,44]. The latter is also present on whey film used as the inner part of the laminate with the 2%CS+CSNPs CIN. After application of the 2%CS-CSNPs CIN onto the whey-based laminate, typical peaks from CS and from CIN indicate the presence of all the deposited formulations at the inner side. Thus, it can be inferred from the bands that it appears on the whey-based laminate after 2%CS+CSNPs CIN application (Figure 3b).

The spectrum for the pure rosemary is shown in Figure 3c (labelled in red) and exhibits typical functional groups known for this kind of phenolic compound [45]. The band at around 1455 cm^−1^ for pure rosemary corresponds to the C-O stretching vibrations of amine and to the C-C stretching from the phenyl group [46]. The same band can be also distinguished at the whey-based laminate when 2%CS+CSNPs ROS is applied, clearly revealing the presence of these compounds on the inner part of the laminate. Despite the fact that many functional groups of pure rosemary and whey-treated film (as the inner layer of laminate) overlap, some characteristic bands from rosemary in the range of 1250–1500 cm^−1^ are also visible from the IR spectra of the whey-based laminate-2%CS+CSNPs ROS.

Interestingly, it was observed that the broad band of chitosan that corresponds to overlapping O-H and N-H vibrations in the case of the whey-based laminate modified with the prepared formulations shifts to higher frequencies in both cases (Figure 3b,c). In the case of the whey-based laminate-2% CS + CSNPs CIN it moves from 3364 to 3412 cm^−1^ (Figure 3b), while in the case of the whey-based laminate-2% CS +CSNPs ROS it shifts from 3353 to 3372 cm^−1^ (Figure 3c). This fact can be correlated with the hydrogen bonding that causes the shift in IR absorption [47]. The latter can be explained by the fact that the hydrogen bonding of the atoms involved causes different changes in the electron density distribution [48]. This change in the IR spectra suggests that the interaction between CSNPs with embedded extracts and the whey-based laminate is most likely hydrogen bonding. ATR-FTIR spectra clearly demonstrate the successful attachment of 2% CS+CSNPs extracts to the surface of the whey film as the inner part of the constructed laminate, allowing for potential bioactivity in contact with the food.

### 2.3. Goniometry

**Figure 4:**
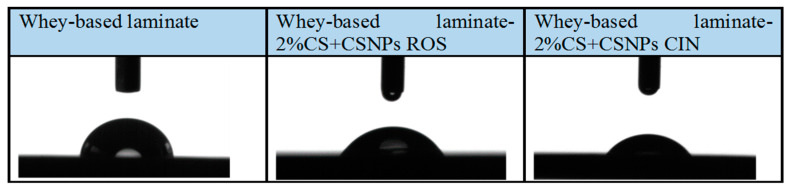
Static contact angle measurements of whey-based laminates.

Based on the static contact angle (SCA) results, the hydrophilicity/hydrophobicity of the whey-based laminate surface was determined. The reduction of the contact angle is of great importance for practical application, as the hydrophilic surface of the packaging material reduces the potential process of condensation (antifogging efficiency) in contact with food. Dew point condensation can significantly worsen packaging conditions and thus increase food contamination [27]. To maintain food quality, it is important to prevent this process. The results of SCA measurements are presented in Table 1 and in Figure 4.

It can be seen that all of the whey-based laminates exhibit hydrophilic character. With the introduction of coatings based on CSNPs with embedded extracts of the “2. Layer”, the contact angle is lowered from 75.63° (whey-based laminate) to around 58°–65°, which from practical point of view is ideal. Additionally, dew condensation is prevented and regulated. Moreover, the wettability of surface is also of a great importance for active interface formation with food, while hydrophilicity and hydrophobicity can also influence interaction with microorganisms. It has also been frequently observed that hydrophilic materials with high surface energy are less susceptible to bacterial adhesion; i.e., it is pointed out that hydrophilic surfaces in contact with media containing organic molecules such as proteins counteract the formation of a conditioning film that harbours adhesion sites for bacteria, which limits the specific adhesion/attachment of bacteria and subsequent biofilm development. One would therefore expect that the hydrophilic character could also contribute to improved antimicrobial activity.

### 2.4. Morphology by SEM

**Figure 5:**
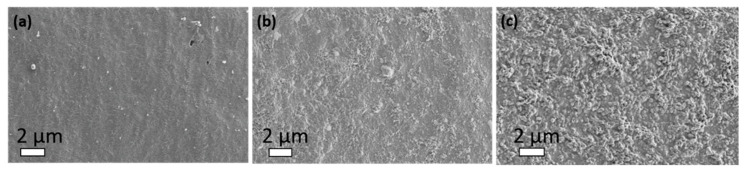
Scanning electron microscopy (SEM) images of the pristine whey protein-based layer as the inner layer of the laminate (**a**); the whey-based laminate treated with 2% CS+CSNPs CIN (**b**); the whey-based laminate treated with 2% CS+CSNPs ROS (**c**). Note that all the images are taken at 10,000× magnification for easier comparison.

To take a closer look to whey-based laminate film morphology before and after treatments, and to clearly reveal the presence of deposited layers, scanning electron microscopy (SEM) images were taken and compared at the magnification of 10,000×. The representative SEM images are shown in Figure 5. The morphology of the inner side of the pristine (uncoated and untreated) whey-based laminate shows interesting morphology for the whey-protein based layer. The surface of the untreated, whey-based layer is smooth and has a special pattern in the form of needles, but some lighter particles can be observed, possibly resulting from the process required to prepare the film for SEM observation, or from the possibility that the untouched film was previously contaminated (Figure 5a). In contrast to the pristine whey-based laminate, the appearance of the deposited layers can be clearly seen for the whey-based laminate functionalised with the 2%CS layer (as the first layer) and extract-embedded CSNPs (Figure 5b,c) as the upper layer. In the case of the whey-protein-based film as the inner part of laminate covered with 2%CS + CSNPs CIN, the rough coating is visible with some particles with a size of less than 1 μm and with a high degree of heterogeneity. It can also be observed that the formed nanoparticles (NPs) are somehow embedded in the first layer of the 2%CS (Figure 5b). Nevertheless, the whey-based laminate film as the inner part is evenly covered with the formulations used, which is of utmost importance from a practical point of view.

Similar but even rougher morphology was obtained for the whey-based laminate film deposited with the 2%CS + CSNPs ROS layer (Figure 5c). The upper (second) layer of the CSNPs with encapsulated ROS is more clearly visible, with quasi-spherical particles ranging well below 1 µm and a more homogeneous formation than in case of the CSNPs CIN. Moreover, in comparison with the whey film with 2%-CS + CSNPs CIN, the density of the deposited NPs is larger here. The latter is in accordance with the dynamic light scattering measurements where higher intensity of CSNPs ROS particles with lower hydrodynamic diameters and with narrower size distribution in the particle range was observed when compared with CSNPs CIN. In general, the obtained results from the electrokinetic analyses (Figure 1) are in line with the results from the SEM analysis. This is inferred from the nanosized CSNPs with both encapsulated extracts. Finally, the SEM pictures clearly show the even coverage of the inner whey-based laminate film with the applied active substances layers. This is highly valued because of the improved antioxidative and antimicrobial properties of the film.

### 2.5. Oxygen Permeability

The permeability of gases, water vapour and organic vapour is of a crucial importance in food packaging applications where the contamination from the environment has to be avoided in order to increase food safety and quality, and to prolong shelf-life. Table 2 contains the results of oxygen transmissions rates (O_2_GTR) and standard deviations (STDV O_2_GTR) for the reference whey-based laminate compared to the functionalised laminates.

The results show that whey-based laminate itself already has extremely low oxygen permeability, comparable to conventionally used barrier layers. It is known that in some food packing applications, non-renewable materials, such as the synthetic barrier EVOH, have already been replaced with whey-based coating due to its similarly good barrier properties and its additional advantage of biodegradability [46]. After the application of coatings to films, the oxygen permeability deteriorated slightly in all cases. It could be seen that in the case of the whey-based laminate + 2%CS + CSNPs ROS, the oxygen permeability is about 7-times higher than in the case of the whey-based laminate with CIN-based formulations. The possible reason for this correlates with the results of the SEM analysis (Figure 5). Namely, the CSNPs ROS are obviously visible on the whey-based laminate, and have a larger active surface area for oxygen permeation due to the larger surface to volume ratio, whereas in the case of the whey treated with CSNPs CIN, most of the particles were embedded into the first applied chitosan layer (Figure 5b,c), and therefore have a smaller active area for oxygen transfer. However, for both functionalised films the results are still great and express a very low oxygen permeability. Permeability of commercially used PE and PP films is 3226 ± 62 cm^3^/m^2^d and 1078 ± 36 cm^3^/m^2^d, respectively, and after a similar concept of functionalization of PE and PP films using chitosan-based formulations, the oxygen permeability was reduced to only 195 cm^3^/m^2^d in the best case [42]. Here, all of the results were much lower, and in the worst case, i.e. whey-based laminate + 2%CS + CSNPs ROS, the oxygen transmission rate is about 85 cm^3^/(m^2^24h). In our opinion, the permeability deteriorates after the application of coatings, while the wettability of whole laminates occurs, causing the structure to relax and consequently the barrier capacity to decrease. In fact, the whey films should be functionalised alone and then integrated into the laminate structure.

### 2.6. Antimicrobial Activity

**Figure 6:**
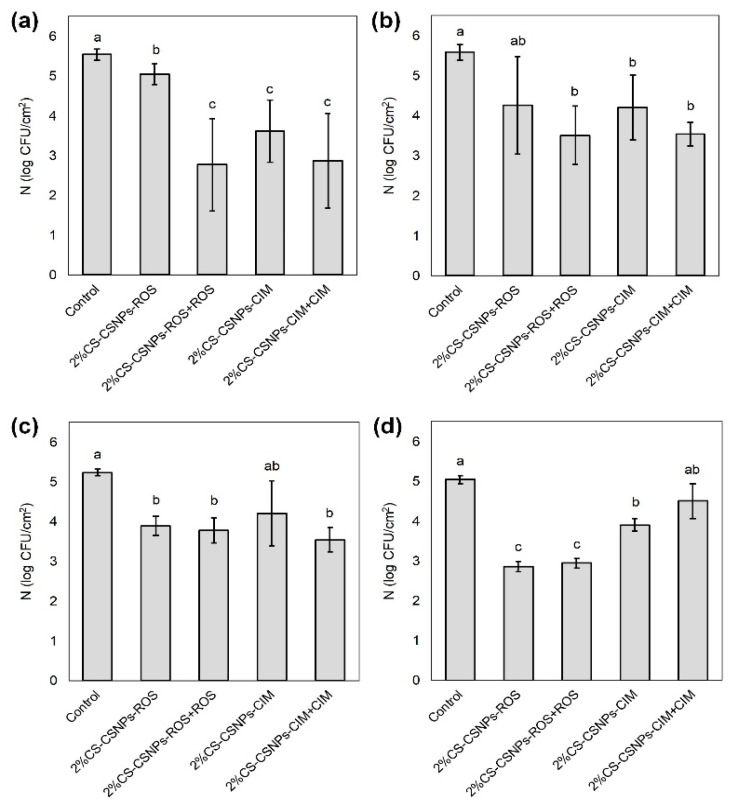
Antimicrobial activity of functionalised whey-based laminates against *Staphylococcus aureus* (**a**), *Escherichia coli* (**b**), *Aspergillus flavus* (**c**) and *Penicillium verrucosum* (**d**). Different letters indicate significant difference (*P* < 0.05).

In Figure 6, the antimicrobial efficacy of pristine and functionalised whey-based laminate is presented. The following microorganisms were tested: Gram-positive bacteria *Staphylococcus aureus,* Gram-negative bacteria *Escherichia coli,* and fungi *Aspergillus flavus* and *Penicillium verrucosum.*

For all of the tested microorganisms, it is clearly shown that most of the functionalised whey-based laminate films have a certain antimicrobial efficacy (*P* < 0.05) compared to the control (only whey-based laminate), where no inhibition was observed. For both bacteria, 2%CS-CSNPs + ROS showed the least effect on antimicrobial activity, and the mean values are lower for the achieved activity of functionalised whey-based laminate films with additional third layer compared to functionalised whey-based laminate films with two layers, but the difference is significant only for *S. aureus* in ROS-functionalised films. Despite the indicated trend in antibacterial activity, this cannot be said with certainty due to the higher standard deviations, which are probably a result of the less homogeneous coating application on this sample. However, this additional third layer of ROS or CIN could improve the antibacterial activity. This could indicate that extracts are the driving force for bacterial inhibition. This has also been observed in *A. flavus* on the CIN-functionalised films, but not in *P. verrucosum*. For both bacteria the antimicrobial activity between ROS and CIN was comparable, but for fungi a better activity was observed in ROS. However, it has been shown in the past for PP and PE, that the highest inhibition is achieved when a synergistic activity of protonated amino groups of chitosan and polyphenols is guaranteed.

These functionalised whey-based laminate films showed antimicrobial activity against bacteria and fungi, which is a highly desirable feature as a wide range of microorganisms which can be found in food can cause spoilage or present a risk to human health. This good antimicrobial activity is most probably a result of the synergistic effect of chitosan and polyphenols [49,50]. Although many publications deal with the antimicrobial activity of chitosan, the exact mechanism is not yet fully understood, and is probably a result of various factors, as it was shown that its antimicrobial activity depends on microbial factors (species, cell age), its intrinsic factors (molecular weight, concentration, positive charge density, hydrophilic/hydrophobic character, chelating properties), physical state (water soluble or solid state) and environmental factors (pH, temperature, ionic strength, exposure time [51]. Based on this, three modes of action of chitosan were proposed: (i) electrostatic interaction between positively charges chitosan molecules and the surface of negatively charged microorganisms, which leads to impairment of the permeability of the membrane wall and causing intracellular components to leak out, subsequently provoking the osmotic imbalance which leads to death of the microorganism [49]; (ii) binding of chitosan to microbial DNA after it enters microbial cells and leads to the inhibition of mRNA and protein synthesis [52]; (iii) chelation of metals to inhibit the growth of essential nutrients for microbial growth [49]. However, currently the most acceptable mechanism is the disruption of cell membranes. It is assumed that CSNPs in our case have a better activity than the microbial cell wall and can be firmly bound to it due to their nanosize and larger surface area [53], and on other hand they can also penetrate the cell wall due to their small size. The desorption profile showed that some chitosan is released and some retains bound to the interface, so both mechanisms are acceptable.

Besides active compounds bound to whey films, the antimicrobial character also depends on the surface chemistry of the composite materials, such as hydrophobicity, amount of charge, available amino groups on the surfaces, roughness, etc. From the above results, it was observed that the sample 2%CS + CSNPs CIN has a lower contact angle and higher band intensities (ATR-FTIR) for amino groups compared to the 2%CS + CSNPs ROS. This may indicate that the amount of amino groups and hydrophilicity may play a slightly more important role in inhibiting bacteria than fungi. Furthermore, it also depends on the roughness and coating homogeneity, which is similar for both samples, however, an even rougher morphology was obtained for the whey-based laminate applied with the 2%CS + CSNPs ROS layer (Figure 5c). It appears that higher roughness is more correlated with fungal inhibition than bacterial inhibition.

### 2.7. Antioxidative Activity

**Figure 7:**
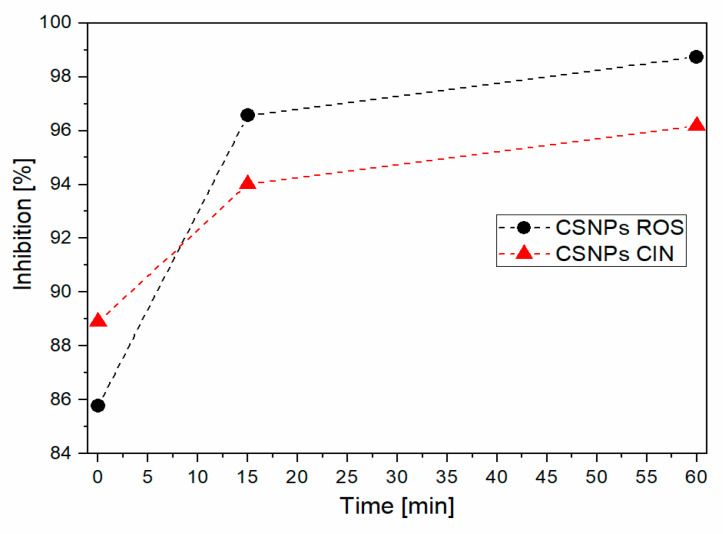
Antioxidative activity of CSNPs dispersions.

**Figure 8:**
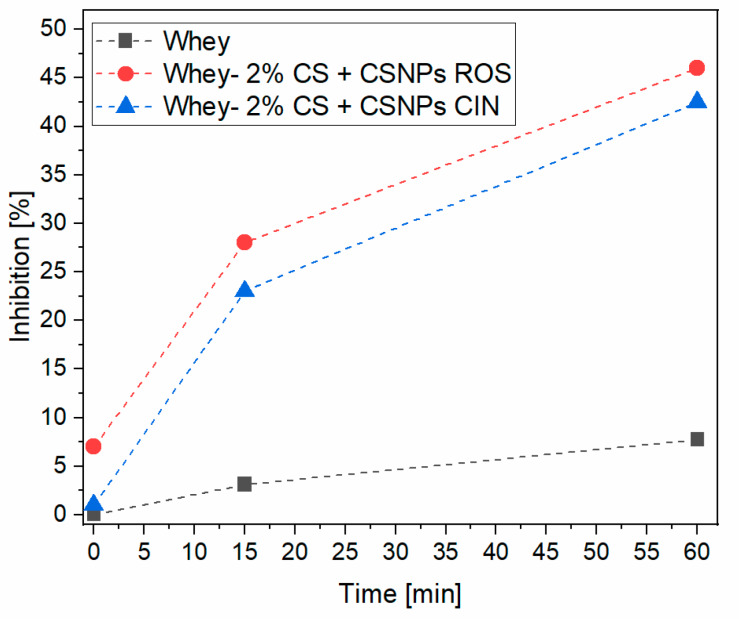
Antioxidative activity of functionalised whey-based laminates.

Figure 7 shows the results of the antioxidant activity for liquid formulations; i.e. CSNPs ROS and CSNPs CIN. It is clearly seen that both formulations have a high antioxidant activity (more than 90% inhibition) established after 15 minutes. When these formulations were applied as a coating onto whey laminate films, as shown in Figure 8, more time was needed to efficiently influence the oxidative process inhibition, mainly due to the release process. It is also observed that pristine whey-based laminate has no antioxidant capacity. After 1 hour of testing, the coatings maintain a quite good antioxidative capacity of around 40% for whey-based laminate+2%CS + CSNPs CIN and 5% for whey-based laminate + 2%CS + CSNPs ROS.

For each coating applied to a whey-based laminate film, extracts must be loaded more successfully (in higher concentrations) into chitosan nanodispersion to obtain an even higher antioxidant capacity, while chitosan itself has relatively poor antioxidant properties [54]. A higher concentration of the extracts can be helpful to achieve an efficiency of around 90%. However, the introduction of approx. 40–50% of the antioxidant capacity is a very good result for whey films, which represent inner films in laminates that are in contact with the packaged substrate and can thus positively influence the shelf-life and reduce food spoilage. In order to obtain an even better bioactive profile of the inner part of the laminate (i.e. for whey films), the technology needs to be optimised.

The following method was also used to estimate the desorption of formulation from the laminate surface. The functionalised laminate was immersed in a desorption bath of distilled water for 24 hours and the reaction between the bath and free radicals was further analysed. The antioxidant activity of both desorption baths was calculated after 60 min of measurement ≈(10–15)%, which means that certain amount of the formulation was desorbed. When calculated into mg, the desorbed amount in relation to the dm^2^ of the laminate migration profile was in accordance with the required overall migration limit (OML) of a compound of surfaces, which according to the guidelines on (European Union (EU)) 10/2011 [40] must be less than 10 mg/dm^2^ or 60 mg/kg food. 

## 3. Materials and Methods

### 3.1. Materials

Low-molecular weight (LMW) chitosan (50 to 190 kDa), deacetylated chitin, Poly (d-glucosamine) from Sigma-Aldrich (Vienna, Austria); sodium tripolyphosphate (TPP, MW = 367.85 g·mol^−1^) from Acros Organics, Geel, Belgium; acetic acid (MW = 60.05 g·mol^−1^), ≥ 99.8% from Sigma-Aldrich; ethanol (MW = 46.07 g·mol^−1^), 99.8% (GC) from Honeywell Sigma-Aldrich; ABTS (2,2’-Azino-bis(3-ethylbenzothiazoline-6-sulfonic acid) diammonium salt) from Sigma-Aldrich; potassium persulfate from Sigma-Aldrich; Milli-Q water Direct system, 0.2 μm PES High Flux Capsule Filter; multilayer laminate consisting of PE, PET and whey from Tuba Lajovic d.o.o., Ljubljana, Slovenia; cinnamon bark; rosemary leaves.

### 3.2. Extraction of the Plant Material

Natural extracts were provided from cinnamon bark and rosemary leaves using two different extraction methods, Soxhlet and cold solvent extraction, respectively.

Soxhlet extraction: 20 g of ground material was first dried and crushed to provide a greater surface area and then poured into a flask. Following this, 150 mL of solvent (EtOH) was added. The flask was then placed in an ultrasonic bath for about an hour. After extraction was complete, the obtained extract solution was filtered and collected in a preweighed round-bottom flask. Finally, the solvent was evaporated under vacuum at 40 °C to collect the extract.

Cold solvent extraction: The powdered materials (20 g) were mixed with 150 mL of EtOH at 25 °C and left stirring using a magnetic stirrer for 4 h. The extracts were filtered in order to remove solid particles and after that they were cooled to room temperature and concentrated under vacuum at 40 °C.

### 3.3. Preparation of Solutions

1% and 2% (*w*/*v*) chitosan aqueous solutions were prepared by dissolving LMW chitosan powder in Milli-Q water. Chitosan dissolution was achieved by adding glacial acetic acid dropwise to the chitosan solution during constant magnetic stirring. The pH was adjusted to 4. Solutions were left stirring during the night until homogeneous dispersions were obtained.

Rosemary and cinnamon extracts, prepared as pointed out in description of extraction procedure, were dissolved in absolute ethanol. The concentration of the extract was determined according to minimal inhibitory concentration (MIC). To ensure the best possible antimicrobial efficacy, the final concentration of the extract was 4× the determined MIC. For this purpose, 20 mg/mL cinnamon and rosemary solutions were prepared. As a crosslinking agent, sodium tripolyphosphate (TPP) was used. TPP powder was dissolved in Milli-Q water, the concentration of the solution was 0.2 wt% in order to obtain 1:5 TPP to chitosan ratio (optimal ratio according to previously published papers) [42,55].

CSNPs embedded with rosemary and cinnamon extract were synthesized by the ionic gelation technique. 20.0 mL of TPP solution and 10.0 mL of extract solution were added dropwise to 20.0 mL of 1 wt% CS solution during stirring. The solution was submitted to continuous stirring for 1 h at room temperature. pH of the solution was adjusted to 4.0 using acetic acid.

### 3.4. Functionalization of the Laminates

The whey-based laminate was first cleaned and dried. Functional coating was applied onto whey-based laminate in two layers; for “1. Layer”, a chitosan macromolecular solution (2%) was applied, and for “2. Layer”, an additive formulation of CSNPs with the incorporation of a rosemary or cinnamon extract was attached (layer-by-layer composition). The roll-to-roll printing method was used for the application of coatings, using a Johannes Zimmer machine, Austria. After each layer, the laminate, based on whey protein film as its inner part, was dried at room temperature. The chitosan macromolecular solution was applied as “1. Layer” due to its high antibacterial efficacy and for better adhesion of CSNPs-extracts dispersions, which hypothetically should exhibit high antioxidant and antifungal activity. In Figure 2 the structure of functionalised whey protein-based laminate is presented. The PE and PET layers are interconnected using an adhesive. The inner side should be in contact with the food. Sample description is given in Table 3.

### 3.5. Electrokinetic Properties and Particle Size

The electrokinetic properties of dispersions were performed with the Zetasizer Nano ZS (Malvern Instruments, Worcestershire, UK) equipped with a He-Ne laser (λ= 633 nm) at a temperature of 25 °C. The signal for dynamic light scattering (DLS) was detected at 173° while for the electrophoretic mobility measurements at 13°. Prior to the measurement, an individual sample was injected into a disposable cuvette (filled up to 1 cm) to measure the hydrodynamic diameter, while for the zeta potential measurements, the sample was injected into a folded capillary cell. Before conducting the analysis, the prepared colloidal formulations were well stirred, and pH was adjusted to 4 using the acetic acid. Finally, the obtained data were collected using the manufacturer’s Zetasizer Software version 7.12 (Malvern Instruments, Worcestershire, UK).

### 3.6. ATR-FTIR Spectroscopy

The changes in the surface functional groups of whey-based laminate and functionalised whey-based laminates were monitored with the ATR-FTIR spectroscopy. ATR-FTIR spectra of samples were performed with the Perkin Elmer Spectrum GX NIR FT-Raman spectrometer (Waltham, MA, USA) and the latter contained a diamond crystal attachment. In order to perform the measurement, the dry whey-based laminates; nonfunctionalised and functionalised, were placed on a diamond crystal and pressed. The corresponding spectra were recorded in the range 400–4000/cm, using 16 scans and at resolution of 4/cm and at room temperature. Prior to the sample measurement, the background spectra were measured using the same parameters. At the end, all the recorded spectra were deconvoluted with baseline correction and smoothing filter. The three repetitions were done for each sample.

### 3.7. Goniometry

The estimation of the surface wettability of the pristine and functionalised whey-based laminates was done by measuring the contact angles using the goniometer DataPhysics, Germany. By using the drop of liquid resting on the surface, the static contact angle was measured. The static contact angle was determined at room temperature with a small drop (3 μL) of ultra-pure Milli-Q water using a goniometer with static contact angle 20 software. The small drop was carefully placed on the surface of the whey-protein-based films, and three repetitions were measured for each film.

### 3.8. Morphology

For SEM imaging of surface morphology and for the distinguishing successful layer deposition, samples of pristine and functionalised whey-based laminates were prepared by cutting into small, approximately 0.5 cm × 0.5 cm square pieces. Afterwards, these pieces were attached to the aluminium sample holders with an adhesive carbon tape in order to ensure conductivity. For this purpose, a Carl Zeiss Supra 35VP scanning electron microscope was employed, where the pristine whey-based laminates and functionalised ones were analysed at an accelerating voltage of 1 kV and variable working distance using 30-µm-sized aperture. All the images were taken at the 10,000 × comparable magnifications.

### 3.9. Oxygen Permeability

An oxygen transmission rate system PERME® OX2/230, Labthink Instruments Co., Ltd PR China was used for the measurement of the oxygen transmission rate (OTR). The measurements were conducted according to the GB/T 19789-2005 or ISO 15105-2:2003 standard. The whey-based laminate thickness was ensured by the producers, as this thickness was used in corresponding measurements. To obtain reliable results, two tests of five measurements by changing the position of measurement (chamber) were performed and expressed as OTR and coefficient values. The measuring conditions for the OTR were 23 °C and 50% relative humidity (flux = 10 mL·min^−1^).

### 3.10. Testing for Antimcirobial Activity

All the microorganisms for the purpose of testing the antimicrobial activity were from the culture collection of the Laboratory for Food Microbiology at the Department of Food Science, Biotechnical Faculty Ljubljana, Slovenia (designation ŽM and ŽMJ). The antimicrobial activity against several microorganisms—bacteria *S. aureus* ŽMJ72 and *E. coli* ŽM370, and fungi *A. flavus* ŽMJ25 and *P. verrucosum* ŽMJ23—was tested.

The whey-based laminates’ antimicrobial potential was evaluated by a modified method (ISO22196) which is designed to determine antimicrobial activity on plastic surfaces [56]. After inoculation to test whey-based film, the bacteria were incubated at 37 °C for one day, while the fungi were incubated for one day at 25 °C. After incubation, numbers of viable bacteria and spores were determined with the pour plate method. For bacteria, plate count agar was used, while for fungi, malt extract agar was used. The number of viable bacteria and spores per cm^2^ for each tested whey protein-based film was calculated. Data assumption of normality (Shapiro–Wilk test) was met, but homogeneity of variance was not. Thus, mean values were compared using Welch analysis of variance (ANOVA) and post hoc Games–Howell test (SPSS V23, IBM, North Castle, NY, USA). *P* < 0.05 was considered as statistically significant.

### 3.11. Antioxidant Activity (ABTS Assay)

The ABTS reagent that was used for the determination of the antioxidative activity of whey-based laminates was firstly dissolved in ultra-pure Milli-Q water to a 7 mM final concentration. ABTS radical cation (ABTS+) was produced by reacting the ABTS stock solution with 2.45 mM potassium persulfate solution (final concertation). This prepared solution was left to store in darkness overnight at room temperature. Afterwards, the ABTS+ solution was additionally diluted with the phosphate buffer solution (pH = 7.4) in order to obtain an absorbance 0.700 ± 0.020 at 734 nm. Then, the diluted ABTS+ solution (3.9 mL) was added to 0.1 g of a specific sample or to 0.1 mL of chitosan nanoparticles with embedded extracts. The corresponding absorbance was measured immediately, after 15 min, and after and 1 h. To gain reliable results, the obtained measurements of all whey-based laminates as well as the reference ABTS+ solution were performed in triplicate. Using Equation (1), the % of radical scavenging activity was calculated, where ABS_control_ is the absorbance of the ABTS+ solution in PBS; ABS_sample_ is the absorbance of the sample (remaining concentration of ABTS radical cation in the presence of extract/whey protein-based films).

Inhibition = ((ABS_control_ − ABS_sample_)/ABS_control_) × 100% (1)

## 4. Conclusions

The aim of this research was to evaluate the effect of the chitosan macromolecular solution and chitosan nanoparticles with embedded rosemary and cinnamon extracts as active coatings on the properties of biodegradable whey-based laminate intended for the packaging industry.

The size and electrokinetic measurements of the prepared formulations showed that the formed extract-encapsulated CSNPs were in the nanoscale and exhibited a highly positive zeta potential. The latter is highly valued because of the improved bioactive properties. From the ATR-FTIR spectra, it could be seen that coatings were successfully applied to the surface of whey films. To prove this, the SEM images also revealed the even coverage of the whey-based laminate with the colloidal formulations. In addition, key properties such as hydrophilicity and low oxygen permeability were ensured, as shown by contact angles below 76° and an oxygen permeability of up to 85 cm^3^/(m24h) for all samples, which is of great importance to achieve food quality and safety, as well as to extend shelf-life.

In terms of bioactivity, the introduction of coatings led to better antimicrobial efficacy compared to the control (only whey-based laminate) due to the synergistic effect of protonated amino groups of chitosan and polyphenols. Similarly, with regard to antioxidant activity, coatings maintained a fairly good antioxidant capacity of about 40% for whey-based laminate + 2%CS + CSNPs CIN and 50% for whey-based laminate + 2% + CSNPs ROS after one hour of testing. Moreover, the migration of chitosan and plant extracts from the laminate surface is below the overall migration limit, which is very important from a practical point of view.

In addition to good applicable properties, such as hydrophilicity of the whey film and good barrier properties in terms of reduced oxygen permeability, these functionalised whey films also show some antimicrobial and antioxidant properties as an inner part of the laminate. Therefore, the process for manufacturing such laminates has a great potential for use in various packaging applications, such as the production of packaging tubes for food products. In addition, the method can even be improved by some refinement steps, such as increasing the polyphenol concentration or applying or manipulating additional layers.

## Figures and Tables

**Table 1:** Contact angles of whey-based laminates.

Sample	Average Angle (α/°)	Difference (°)
Whey-based laminate	75.63° ± 1.60°	/
Whey-based laminate-2%CS-CSNPs ROS	65.40° ± 1.30°	−10.23°
Whey-based laminate-2%CS-CSNPs CIN	58.90° ± 1.20°	−16.73°

**Table 2:** Oxygen permeability of whey-based laminates.

Sample	O_2_GTR (cm^3^/(m^2^24h))	STDV O_2_GTR	Thickness (mm)
Whey-based laminate	6.585	0.523	0.4
Whey-based laminate-2%CS-CSNPs ROS	84.667	4.272	0.4
Whey-based laminate-2%CS-CSNPs CIN	12.679	1.047	0.4

**Table 3:** Sample description.

Sample	Description
Whey-based laminate	Laminate consisting of polyethylene (PE), polyethylene terephthalate (PET) and whey protein-based layer (inner part)
Whey-based laminate-2%CS-CSNPs ROS	Whey-based laminate functionalised with 2% chitosan solution (2% CS) for “1. Layer” and chitosan nanoparticles with embedded rosemary extract (CSNPs ROS) for “2. Layer”
Whey-based laminate-2%CS-CSNPs ROS + ROS	Whey-based laminate-2% CS + CSNPs ROS additionally coated with rosemary ethanolic solution
Whey-based laminate-2%CS-CSNPs CIN	Whey-based laminate functionalised with 2% chitosan solution (2%CS) and chitosan nanoparticles with embedded cinnamon extract (CSNPs CIN)
Whey-based laminate-2%CS-CSNPs CIN + CIN	Whey-based laminate-2%CS + CSNPs CIN additionally coated with cinnamon ethanolic solution
